# Computational approach towards the design of artemisinin–thymoquinone hybrids against main protease of SARS-COV-2

**DOI:** 10.1186/s43094-021-00334-z

**Published:** 2021-09-06

**Authors:** Victor Moreira de Oliveira, Matheus Nunes da Rocha, Emanuel Paula Magalhães, Francisco Rogênio da Silva Mendes, Márcia Machado Marinho, Ramon Róseo Paula Pessoa Bezerra de Menezes, Tiago Lima Sampaio, Hélcio Silva dos Santos, Alice Maria Costa Martins, Emmanuel Silva Marinho

**Affiliations:** 1 Theoretical and Electrochemical Chemistry Research Group/FAFIDAM, State University of Ceará, Limoeiro do Norte, CE CEP 62930-000 Brazil; 2 Department of Clinical and Toxicological Analysis, Federal University of Ceara, Fortaleza, CE CEP 60430-172 Brazil; 3 Iguatu Faculty of Education, Science and Letters/FECLI, State University of Ceará, Iguatu, CE CEP 63502-253 Brazil; 4 Laboratory of Natural Products Chemistry, Synthesis and Biocatalysis of Organic Compounds – LBPNSB, State University of Vale do Acaraú, Sobral, CE CEP 62040370 Brazil

**Keywords:** COVID-19, Pharmacokinetics, Main protease, Decarboxylation hybrids

## Abstract

**Background:**

The sanitary emergency installed in the world, generated by the pandemic of COVID-19, instigates the search for scientific strategies to mitigate the damage caused by the disease to different sectors of society. The disease caused by the coronavirus, SARS-CoV-2, reached 216 countries/territories, where about 199 million people were reported with the infection. Of these, more than 4 million died. In this sense, strategies involving the development of new antiviral molecules are extremely important. The main protease (Mpro) from SARS-CoV-2 is an important target, which has been widely studied for antiviral treatment. This work aims to perform a screening of pharmacodynamics and pharmacokinetics of synthetic hybrids from thymoquinone and artemisin (THY-ART) against COVID-19.

**Results:**

Molecular docking studies indicated that hybrids of artemisinin and thymoquinone showed a relevant interaction with the active fraction of the enzyme Mpro, when compared to the reference drugs. Furthermore, hybrids show an improvement in the interaction of substances with the enzyme, mainly due to the higher frequency of interactions with the Thr199 residue. ADMET studies indicated that hybrids tend to permeate biological membranes, allowing good human intestinal absorption, with low partition to the central nervous system, potentiation for CYP-450 enzyme inhibitors, low risk of toxicity compared to commercially available drugs, considering mainly mutagenicity and cardiotoxicity, low capacity of hybrids to permeate the blood–brain barrier, high absorption and moderate permeability in Caco-2 cells. In addition, T1–T7 tend to have a better distribution of their available fractions to carry out diffusion and transport across cell membranes, as well as increase the energy of interaction with the SARS-CoV-2 target.

**Conclusions:**

Hybrid products of artemisinin and thymoquinone have the potential to inhibit Mpro, with desirable pharmacokinetic and toxicity characteristics compared to commercially available drugs, being indicated for preclinical and subsequent clinical studies against SARS-CoV-2. Emphasizing the possibility of synergistic use with currently used drugs in order to increase half-life and generate a possible synergistic effect. This work represents an important step for the development of specific drugs against COVID-19.

## Background

Coronavirus Disease (COVID-19), caused by the severe acute respiratory syndrome coronavirus 2 (SARS-CoV-2), is responsible for causing acute respiratory incapacity, which, if left untreated, can result in death [1–3]. The pathology first spread in Wuhan, China, and then became a world emergency and the start of a pandemic and threat to public health on 12/31/2019, according to the World Health Organization (WHO) [4]. Structurally, SARS-CoV-2 possesses a single strand of positive sense RNA as genetic material, which encodes structural capsid proteins, in addition to enzymes.

In this context, among different pharmacological targets currently being studied, the main protease (Mpro) stands out, which plays a fundamental role in viral replication. Mpro enzyme is activated after its autocleavage, generating two subunits [5]. Theoretical studies have identified a region of inhibition of the active fraction of the enzyme, characterized by the formation of a complex between Mpro and the ligand N3. This finding has contributed to the prospection of new drugs in screening in silico [6].

Artemisinin (ART) is named by many authors as a gift from traditional Chinese medicine to the world. It is the major component of a plant called *Artemisia annua* and has been applied in the treatment of malaria and other diseases caused by protozoa from *Plasmodium* sp. genus [7]. Thymoquinone (THY) is the most abundant constituent of *Nigella sativa* oil, characterized by having several pharmacological properties, such as anti-cancer, gastroprotective, hepatoprotective and nephroprotective, all associated with its anti-oxidative, anti-inflammatory and immunomodulatory potential [8].

It has been previously described that ART and THY possess antiviral activity [9]. Recently, it was reported that bioactive compounds present in *Nigella sativa* seeds, including thymoquinone and dythimoquinone, were able to interact with SARS-CoV-2 spike protein:ACE2 receptor interface [10]. THY also interacted with Mpro on in silico simulations [11]. Additionally, ART and its derivatives presented cardioprotective effect in COVID-19 over ACE2 signaling pathway [12].

However, ART and THY present considerable toxicity, which can lead to hypoactivity and difficulty in breathing [13], as well as neurotoxicity, embryotoxicity, genotoxicity, hemato and immunotoxicity, cardiotoxicity and allergic reactions [14]. The potential of thymoquinones to increase the antioxidant capacity of mice mesenchymal stem cells has also been described in the literature, favoring their migration and inducing immunogenicity in vivo [9]. In order to maintain or increase their pharmacological properties and, concomitantly, reduce their toxicity, hybridization techniques can be used, aiming the development of compounds with better bioavailability and less toxic risk [15–17].

Therefore, the present study selected a series of molecules previously reported by Fröhlich et al. [17], which consists of hybrids of THY, ART and its derivative artesunic acid (ARA) [9]. These compounds were synthesized by structural modification of artesunic acid, through the addition of thymoquinone by decarboxylation. Previously, some of these molecules demonstrated antimalarial activity and effect against Human Cytomegalovirus (HCMV) in vitro. Therefore, this work aims to perform a theoretical screening of pharmacodynamics and pharmacokinetics of these molecules on SARS-CoV-2 main protease (Mpro).

## Methods

### Computational details

All simulations were performed using free codes for academic use in a 64-bit operating system. The codes were used: Pymol [18], UCSF Chimera™ [19], Autodocktools™ [20], AutoDockVina™ [21], Avogadro™ (http://avogadro.cc/) [22], Discovery studio visualizer™ viewer [23] e Marvin™ 19.8, 2020, (http://www.chemaxon.com) [24].

### Obtaining and optimizing molecular structures

**Fig. 1:**
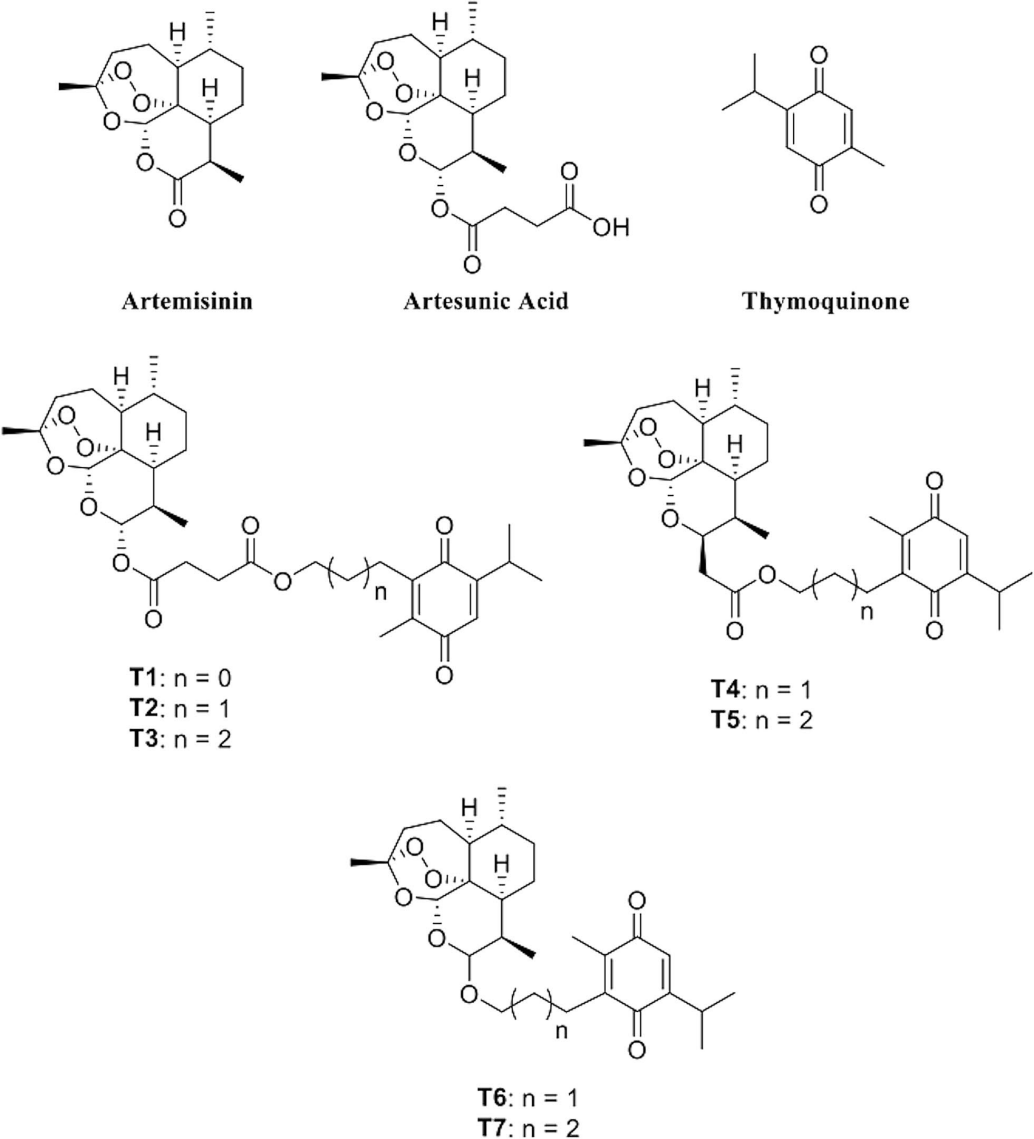
Structural formula of parental ligands artemisinin, artesunic acid and thymoquinone and synthetic hybrids from artemisinin–thymoquinone T1–7. Adapted from Fröhlich et al. 2018 [17]

**Fig. 2:**
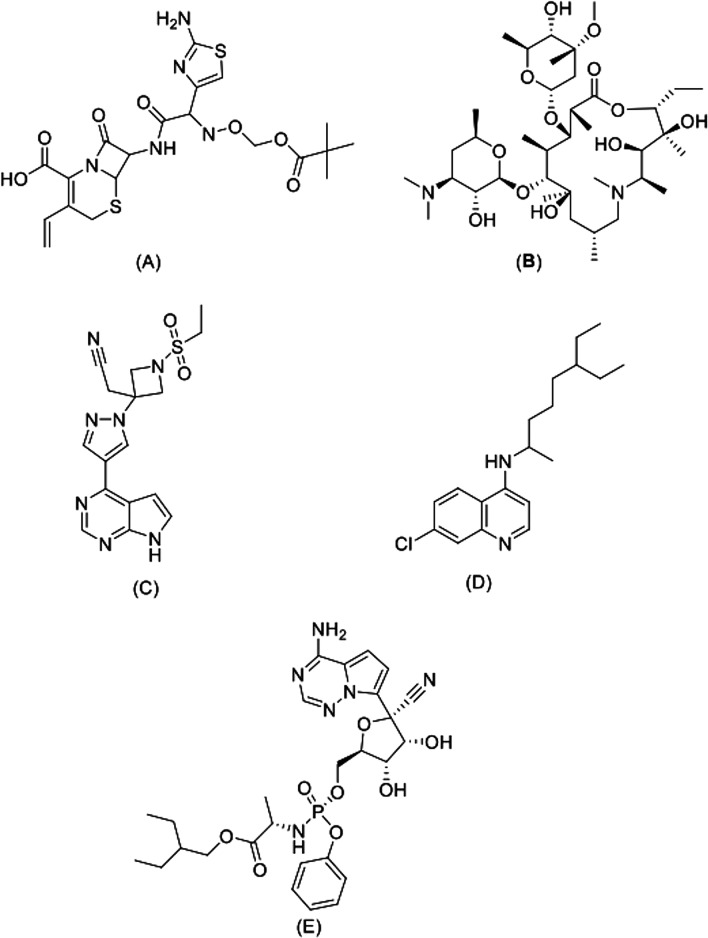
Anti-SARS-COV-2 structural formula drugs used as control ligands. **A** Anakinra, **B** azithromycin, **C** baricitinib, **D** chloroquine e **E** remdesivir. Adapted from PubChem

The molecular structures of THY, ART, ARA and hybrids (T1–T7) were obtained from the study by Fröhlich et al. [17] (Fig. 1). As control ligands, anakinra (ANK) (PubChem CID: 139595263), azithromycin (AZT) (PubChem CID: 447043), baricitinib (BRT) (PubChem CID 44205240), chloroquine (CLQ) (PubChem CID: 2719) and remdesivir were used (RDS) (PubChem CID: 121304016) (Fig. 2). In addition, the results were compared to those obtained for the enzyme inhibitor, peptide N3 (PRD_002214), which is covalently bound with Cys145 residue [25].

The molecules were designed using the MarvinSketch^®^ academic license software version 20.13 [24] from the ChemAxon^©^ Marvin software package (https://chemaxon.com/products/marvin). The three-dimensional structure of the selected compounds was optimized using the classic force field method MMFF94 (Merck Molecular Force Field 94) [26], implanted in the free Avogadro^®^ software [22], programmed to perform a cycle of 4 interactions of the Steepest Descent algorithm, following the parameters defined in Eq. (1), where the most stable structure is obtained by minimizing the potential energy (*E*) of a molecule in its steady state, with the contribution of a force (*k*_b_) exerted on a bond between two atoms (*r* − *r*_0_) and the sum includes all bonds of the molecule's three-dimensional space [27].

1 $$ E = \sum k_{{\text{b}}} \left( {r - r_{0} } \right)^{2} $$

### General Docking procedures

The crystallographic structure of the Mpro enzyme of COVID-19 conjugated to N3 was obtained from Protein Data Bank (https://www.rcsb.org/), where it was deposited with the PDB ID code 6LU7, and is composed of three domains: domain I (residues 8–101), domain II (residues 102–184) and domain III (residues 201–303), in addition to a long loop (residues 185–200) linking domain II to domain III [6].

Molecular Docking simulations and Re-docking were performed using AutoDockVina Version 1.1.2 [28]. For each analysis, 100 cycles of 10 independent simulations were performed applying Lamarkian Genetic algorithm [22, 23]. Mpro SARS-CoV-2 remaining parameters were set as standard. For Mpro SARS-CoV-2 were used 3-way multithreading and the following parameters: center_*x* = − 26.734, center_*y* = 13.009, center_*z* = 56.185, size_*x* = 94, size_*y* = 112, size_*z* = 108, spacing = 0.642 [29]. Non-protein molecules were removed, and polar hydrogen were added using AutoDock Tools 1.5.6 software (ADT, http://mgltools.scripps.edu/) [30].

Following the methodology proposed by Marinho et al. [31], 50 independent simulations were carried out with 20 poses each using the same Exhaustiveness criteria 8. At the end of the coupling simulations, several ligand binding energies were obtained with their respective conformations; the stable conformation, which corresponds to the lowest energy connection, was chosen as the best pose and was used in the docking analysis. The bonding energies were calculated using Eq. (2).

2 $$G=-RT\mathrm{Ln}K$$

were ∆*G* is the binding free energy in KJ mol^−1^, *R* is the gas constant, 8.32 J mol^−1^ K^−1^ and *T* is the absolute temperature, 298 K.

To validate the simulations, Redocking procedures were performed, and the RMSD (root-mean-square deviation) values were evaluated within the ideal parameter, less than 2 Å [32] affinity energy was used as a parameter, with ideality parameters values below − 6.0 kcal/mol [30].

### Physicochemical, pharmacokinetic and toxicity properties

The molecular mass (MW), rotating bonds (RB), number of H-bond acceptors and donors (HBA and HBD) and the topological polar surface area (TPSA) of the molecules were calculated using MarvinSketch^®^ software version 20.13 (ChemAxon^©^ https://chemaxon.com/products/marvin) [16, 27]. The partition coefficient (log *P*) and water solubility at pH 7.4 (log *S*) were calculated using the preADMET server (https://preadmet.bmdrc.kr/) [33].

Aiming to trace a prediction of the validation of hybrids as candidates for drugs intended for oral administration, the druglikeness criteria of Lipinski's 'rule of 5′ [34], which classifies a compound as a good candidate for oral use that does not violates more than 1 of the following criteria: MW ≤ 500 g/mol, log *P* ≤ 5, HBA ≤ 10 and HBD ≤ 5.

The pharmacokinetics of the compounds were estimated by predictive parameters of absorption, distribution, metabolism and excretion (ADME) at pH 7.4 through the preADMET server (https://preadmet.bmdrc.kr/). The following were evaluated: human intestinal absorption (HIA) [35]; permeability through the intestinal epithelium, using the Caco-2 model [30, 32]; plasma protein binding (PPB) [36]; permeability through the blood–brain barrier (BBB) [29, 33]; and interaction with cytochrome P450 (CYP450) enzymes. The toxicity parameters analyzed were: mutagenicity and carcinogenicity, both using the Ames test [37]; and cardiotoxicity, by inhibition of the hERG potassium channel (Human Ether-a-go-go-Related Gene).

The identification of potentially pharmacophores was performed by testing the similarity of QSAR (quantitative structure–activity relationship) models from the Pred-hERG online server, LabMol (http://predherg.labmol.com.br/), generating a 2D visualization probability map.

## Results

### Molecular docking

**Fig. 3:**
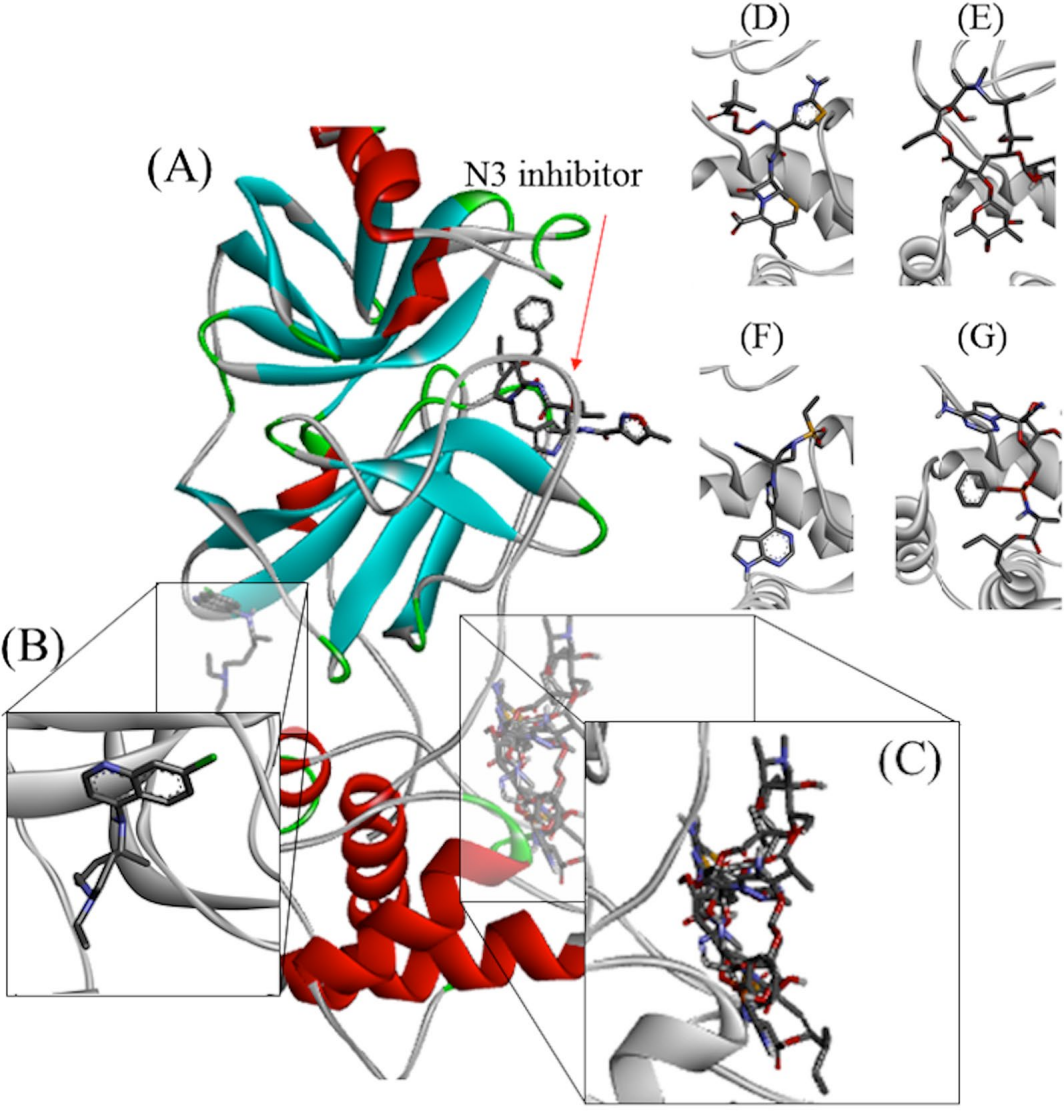
Drug interaction sites used as control ligands identified by molecular docking. In the figure, Mpro SARS-COV-2 is presented docked with **A** N3 inhibitor; **B** chloroquine, **C** control ligands; **D** anakinra, **E** azithromycin, **F** baricitinib e **G** remdesivir

Figure 3 shows the interactions analyzed by molecular docking. When the ligands were compared regarding their interactions with Mpro, it was possible to separate them into two distinct groups (Fig. 3A) (black squares). It was observed that T6 and T7 belong to the same group of AZT, ART and BRT, while T1–T5 belong to the group of RDS, ANK, ARA and THY. It is also noteworthy the presence of false positivity found for the relationship between CLQ and T7, given the distance between the sites of activity of these two ligands. Figure 3B shows the interaction between Apro de Mpro residues to identify conserved residues of importance for enzymatic activity, identified by the orange arrow. Of these, waste K137, T199 and L287 stand out. In addition, it was observed that the residues that interact with BRT and N3 are isolated from the other.

Figure 3C shows the general interaction between ligands and residues, with emphasis on the different interaction sites for each group. Thus, the approximation of the substances T1–T5 to the sites of ANK and RDS is ratified, while T6–T7 approaches the sites AZT and BRT. Once again, this figure shows that CLQ did not interact in a relevant way with the Aspro residues of Mpro, indicating that it is not an inhibitor of this enzyme.

**Fig. 4:**
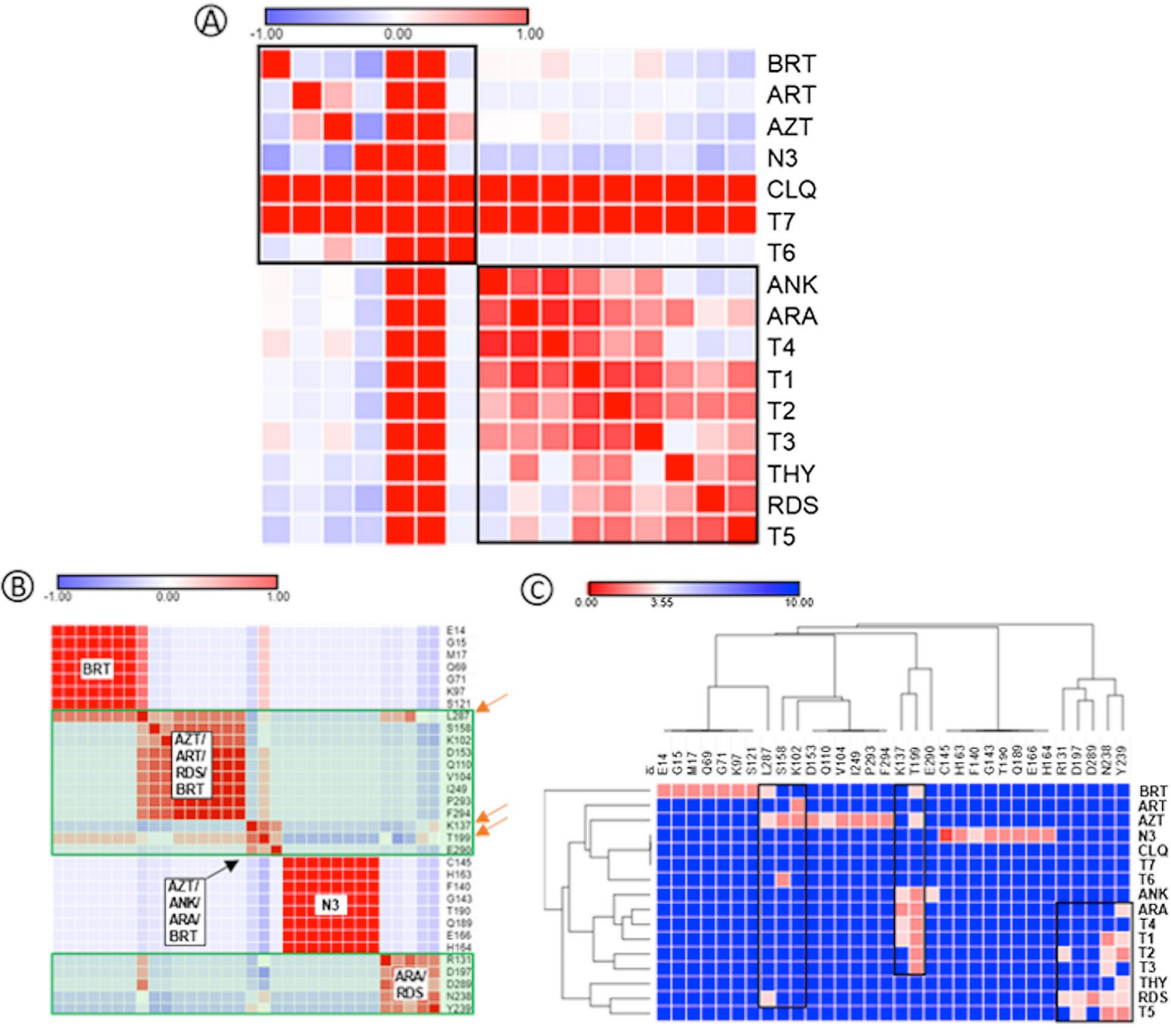
Heatmap representation of binding between Mpro and ligands. **A** Pearson’s similarity test for ligands analyzed on this study; **B** Pearson’s similarity test between Mpro amino acid residues; **C** interactions between ligands and Mpro residues. AZT (azithromycin); BRT (baricitinib); RDS (remdesivir); ANK (anakinra); CLQ (chloroquine); ART (artemisin); ARA (artemisin acid); THY (thymoquinone). For interpretation, the range varies from − 1 (blue) to + 1 (red), where red indicates stronger and closer interactions, while blue indicates weak and distant interactions

The data demonstrate that ANK, AZT, BRT and RDS occupied the same catalytic site of Mpro, in a different site from that occupied by the N3 inhibitor (Fig. 4). ANK, AZT and BRT form regions of strong hydrogen interaction with the Thr199 residue, while RDS forms a region of slightly strong hydrogen interaction with Leu287.

**Fig. 5:**
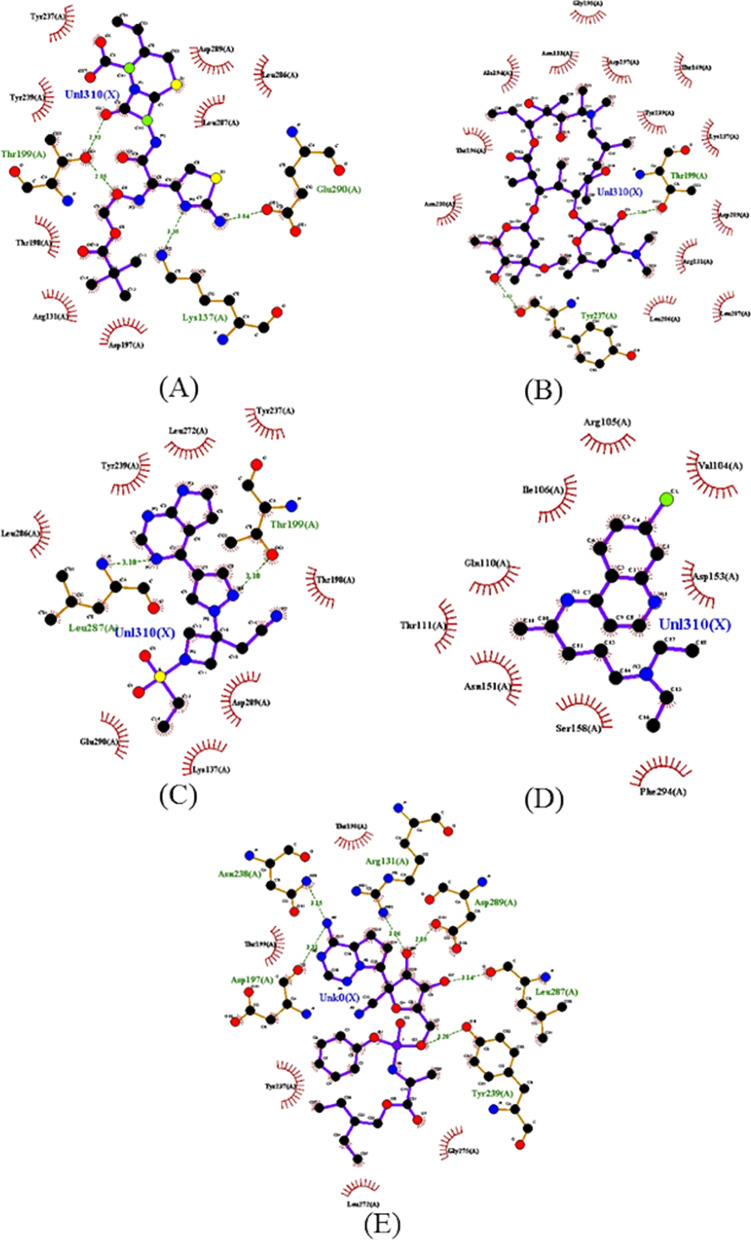
2D map representative of H-bonds between control ligands and Mpro SARS-CoV-2. **A** Anakinra, **B** azithromycin, **C** baricitinib, **D** chloroquine e **E** remdesivir

When hydrogen interactions and bond distances involving control ligands are analyzed (Fig. 5), it is confirmed that CLQ interacts weakly with a site distinct from other substances. Table 1 shows that, among the control ligands, CLQ has the highest interaction energy, in an energy order of − 4.7 kcal/mol.

**Table 1:** Interactions, distances and energy parameters of ligands in molecular docking with Mpro SRAS-CoV-2

Compound name	Δ*G* (kcal/mol)	Hydrogen bonding		
		Atom of ligand	Amino acid	Distance (Å)
*Parental ligands*				
ART	− 6.2	O *sp*^2^	Lys102	2.96
ARA	− 6.7	O *sp*^2^	Arg131	3.02
		O *sp*^2^	Thr199	2.88
		O *sp*^2^	Thr199	3.16
		O *sp*^3^	Asn238	3.12
		O *sp*^2^	Tyr239	2.90
THY	− 4.08	O *sp*^2^	Tyr239	3.11
*Artemisinin–thymoquinone hybrids*				
T1	− 7.6	O *sp*^2^	Lys137	2.96
		O *sp*^2^	Thr199	2.94
		O *sp*^3^	Tyr239	3.15
T2	− 7.5	O *sp*^2^	Lys137	3.04
		O *sp*^3^	Thr199	2.93
T3	− 8.3	O *sp*^3^	Lys137	3.02
		O *sp*^2^	Lys137	3.06
		O *sp*^2^	Lys137	3.29
		O *sp*^2^	Thr199	2.86
		O *sp*^3^	Thr199	3.05
		O *sp*^3^	Thr199	3.17
		O *sp*^2^	Asn238	2.94
		O *sp*^3^	Tyr239	3.08
T4	− 7.7	O *sp*^3^	Thr199	2.88
		O *sp*^2^	Asn238	3.17
T5	− 7.8	O *sp*^2^	Asp197	3.31
		O *sp*^3^	Asn238	2.94
		O *sp*^3^	Tyr239	2.09
T6	− 8.3	O *sp*^3^	Ser158	2.97
T7	− 7.7	–	–	–
*Control-ligands*				
ANK	− 6.2	N *sp*^2^	Lys137	3.35
		O *sp*^3^	Thr199	2.85
		O *sp*^2^	Thr199	2.93
		N *sp*^3^	Glu290	3.04
AZT	− 6.9	O *sp*^3^	Thr199	3.04
		O *sp*^3^	Tyr237	3.85
BRT	− 6.8	N *sp*^2^	Thr199	3.10
		N arm	Leu287	3.10
CLQ	− 4.7	–	–	–
RDS	− 6.8	O *sp*^3^	Arg131	3.06
		N *sp*^3^	Asp197	3.31
			Asn238	3.15
			Tyr239	3.20
			Leu287	3.14
			Asp289	2.85

*AZT* azithromycin, *BRT* baricitinib, *RDS* remdesivir, *ANK* anakinra, *CLQ* chloroquine, *ART* artemisin, *ARA* artemisin acid, *THY* thymoquinone

Additionally, still in Table 1, it is possible to notice that the parental compounds ARA and THY occupy the same catalytic site as most control ligands, highlighting interactions in common with Tyr239 and Thr199 residues. ARA forms a hydrogen bond with the Tyr239 residue, with a strong contribution from the ester group attached to the hexacyclic ring, while THY binds to the residue with contribution from the carbonyl bonded to the unsaturated hexacyclic ring. Furthermore, ARA presents an interaction with Thr199 of similar intensity to that of AZT, which presents a distance of 3.04 Å.

**Fig. 6:**
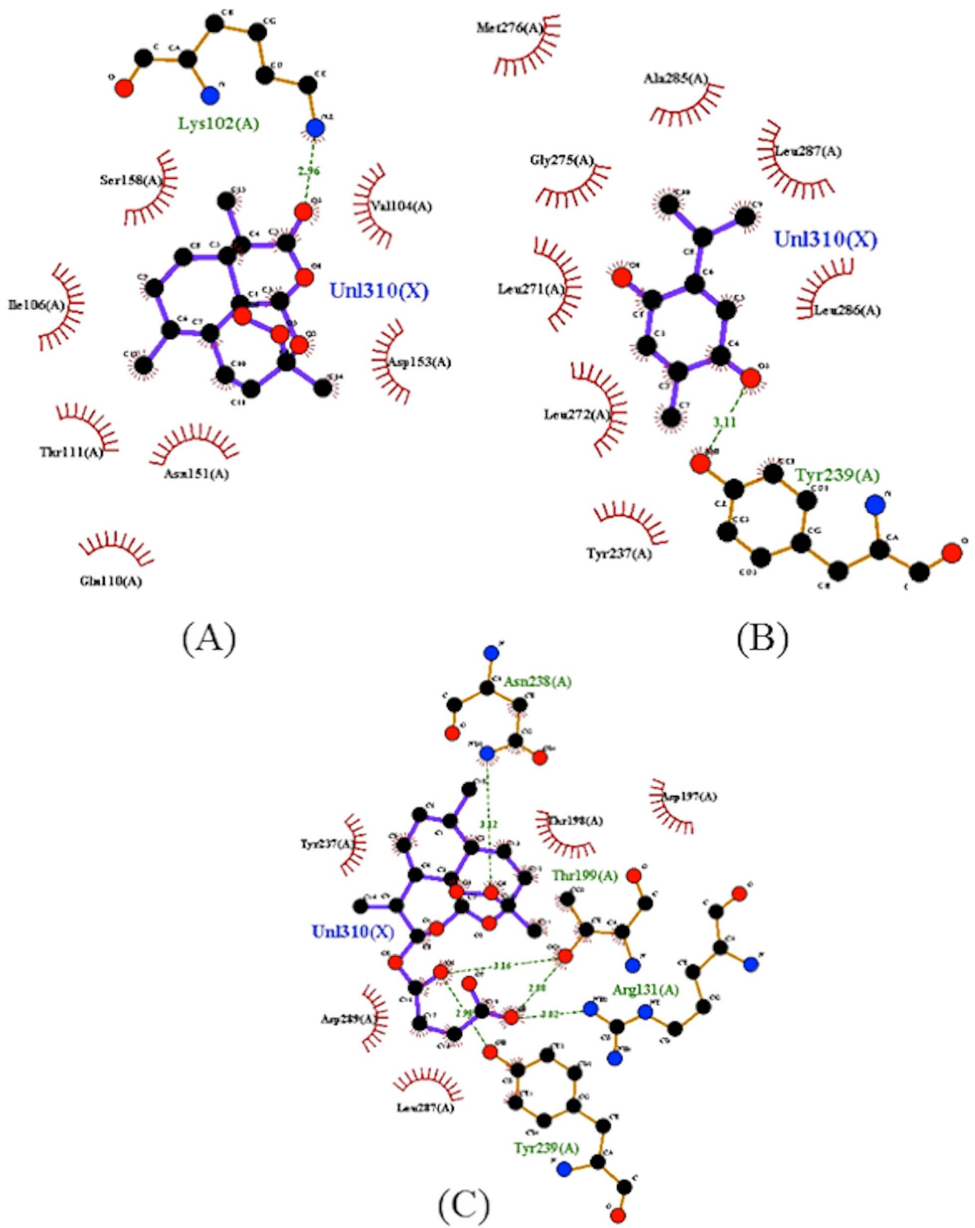
2D map representative of H-bonds between parental ligands and Mpro SARS-CoV-2. **A** Artemisinin, **B** artesunic acid, **C** thymoquinone

In addition, ART interacts with Lys102 through the hydrogen bonding carbonyl receptor connected to the hexacyclic ring. This being the only one among the parental ligands used in the structural modification to interact with this catalytic site, presenting a minimum affinity energy of − 6.2 kcal/mol. The results of the present work demonstrate that the hydrogen bond formed between ART and Lys102 presented a bond distance of about 2.5 Å < *d* < 3.1 Å, which represents a region of strong interaction [38]. In Fig. 6, hydrogen interactions and bonding distances of parent compounds are illustrated, allowing for better visualization.

**Fig. 7:**
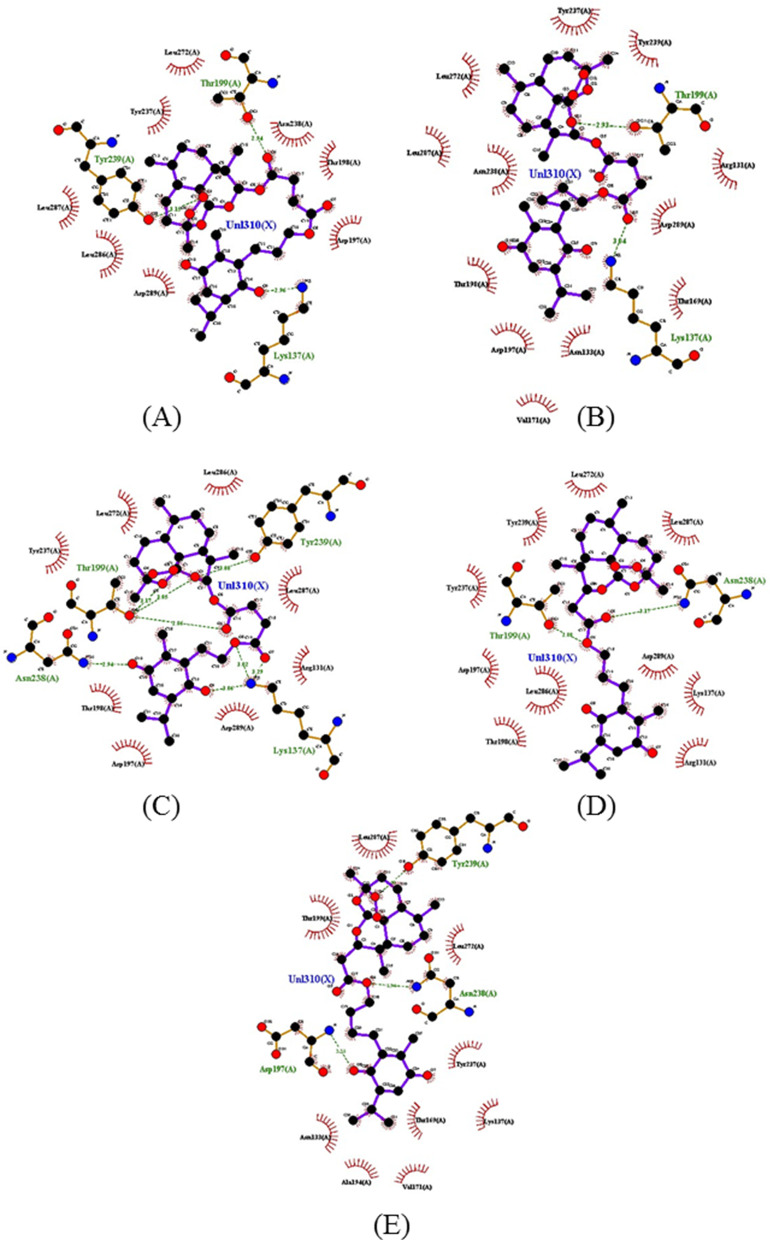
2D map representative of H-bonds between ART-THY hybrids. **A** T1, **B** T2, **C** T3, **D** T4 e **E** T5

When the interactions of the artemisinin–thymoquinones hybrids were analyzed, it was found that they showed improvement in the interaction of the substances with the enzyme, as shown in Fig. 7. The results allow observing the higher frequency of interactions with the Thr199 residue associated with T1–T4 substances, with a strong contribution of oxygen from the hydrogen bonding esters of the ligands T1, T3 and T4, and oxygen from the hexacyclic structure of T2. The T5 ligand, on the other hand, interacts with the Tyr239 residue, which is frequent between the parent ligands artesunic acid and thymoquinone. It is worth mentioning that the substances are bound to the Thr199 residue by hydrogen bonds of the donor-recipient type, where the distances follow an order of 2.5 Å < *d* < 3.1 Å, generating a region of strong interaction and with energy balance that varies from − 8.3 to − 7.5 kcal/mol (Fig. 7) [38].

**Fig. 8:**
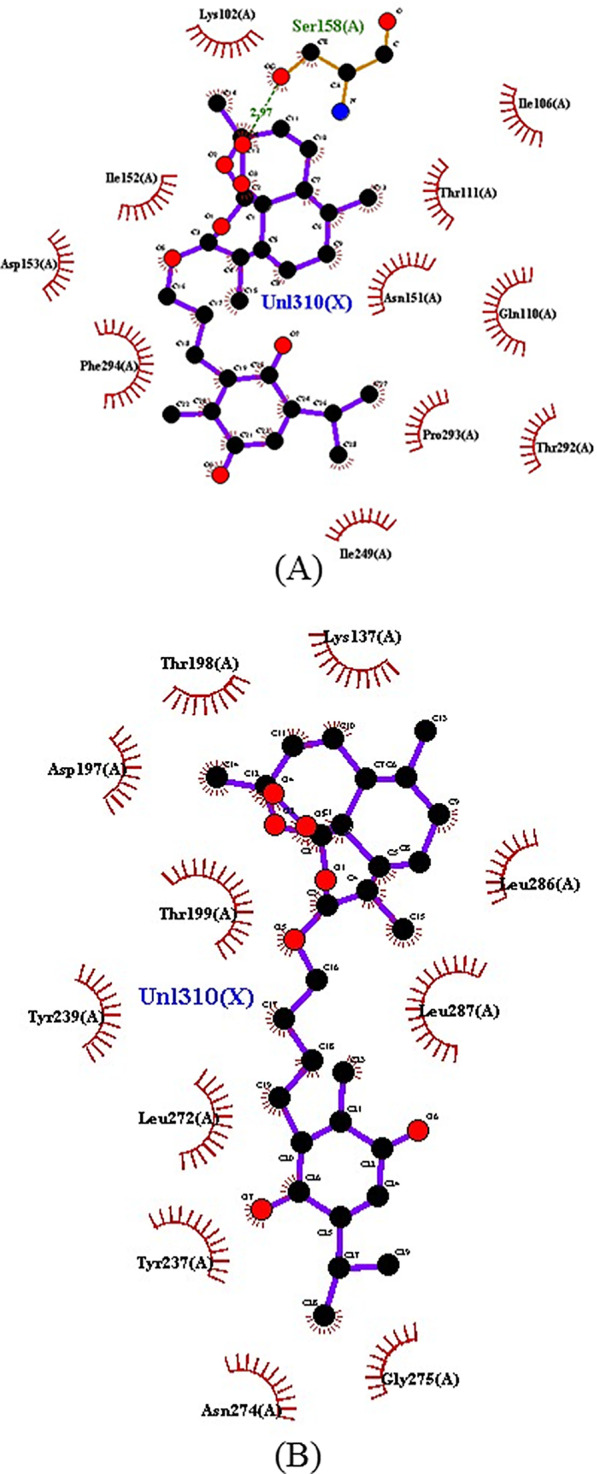
2D map representative of H-bonds between ART-THY hybrids. **A** T6 e **B** T7

**Fig. 9:**
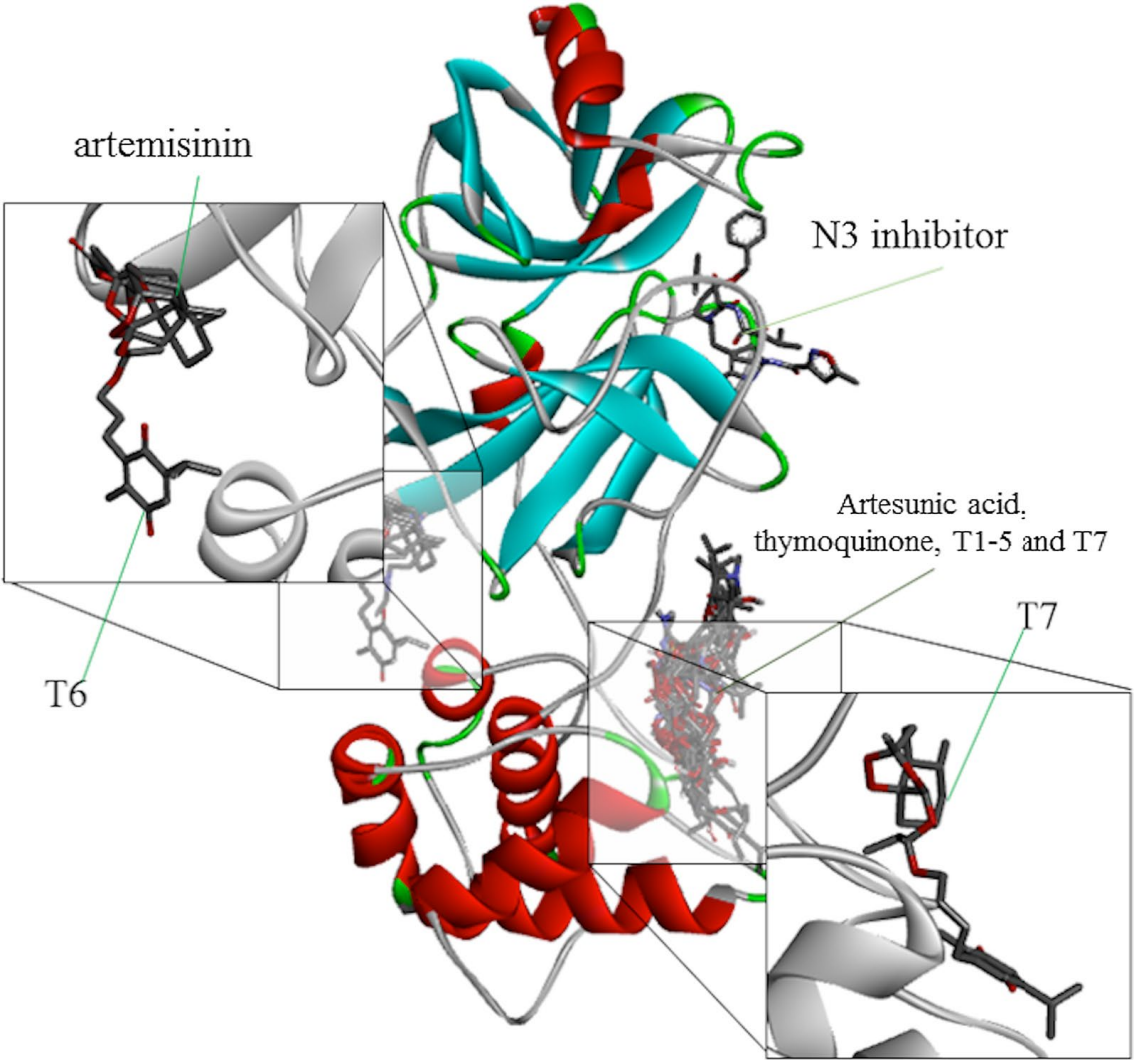
Representation of the interaction of hybrids T6 and T7 as molecules with Mpro SARS-CoV-2

Additionally, the T6–T7 ligands showed peculiarities in comparison with the other hybrids. The T6 ligand is the only one of the hybrid ligands to couple at the same site as chloroquine, interacting with the Ser158 residue by hydrogen bonding, as well as the substance artemisinin (Fig. 6A). This interaction takes place through one of the oxygen grouped to the cyclic structure as shown in the map in Fig. 4. It is possible to highlight the hydrogen interaction of the T6 ligand to the Ser158 residue as a region of strong ligand–receptor interaction, with distance calculated in the order 2.5 Å < *d* < 3.1 Å and the affinity energy evaluated at − 8.3 kcal/mol, which is the lowest among the ligands in the study, resulting in their best thermodynamic conditions of interaction (Figs. 8, 9).

The ligand T7 forms a series of weak interactions at the catalytic site where most of the ligands in this study are found. Probably, the T7 ligand was captured by the catalytic site with binding energy strong enough to maintain it despite its weak individual interactions. Figure 8 illustrates the diversity of these interactions, highlighting T6 and T7 as promising ligands to the enzyme Mpro. The diversity of sites of interaction with the enzyme stands out when comparing artemisinin and T6 with Artesunic acid, thymoquinone, T1–T5 and T7, in addition to the N3 inhibitor, demonstrating that the interactions of parental ligands and hybrids with the enzyme present a pattern similar to that observed by the control ligands.

### Physicochemical and drug-likeness properties

The physical–chemical properties of the compounds analyzed in the present study are listed in Table 2. Based on the criteria defined by the “rule of five” from Lipinski, the T6 ligand was the only hybrid of structural modifications within the physicochemical space defined by the four limits (MW ≤ 500 g/mol, log *P* ≤ 5, HBA ≤ 10 and HBD ≤ 5), while the ligands T2, T5 and T7 obtained a double violation of the type MW > 500 g/mol and log *P* > 5 [34]. However, T1–T7 hybrids are within the physicochemical compound space of Pfizer, Inc., (low relative log*P* and high TPSA), satisfying the pharmacokinetic attributes: high permeability, low risk of passive efflux and low metabolic clearance, as well as safety parameters, ensuring a low toxic risk by oral administration [39] while ANK, AZT and RDS have their oral bioavailability and pharmacokinetic attributes limited by exceeding the physicochemical safe space established by the "rule of five" criteria.

**Table 2:** Physicochemical and pharmacokinetic properties of ART-THY hybrids, parental and control compounds

Compound	MW (g/mol)	N.RB	N.HBAs	N.HBDs	Physicochemical properties				Pharmacokinetics			
					TPSA at pH 7.4 (Å^2^)	Lipophilicity	Water solubility at pH 7.4		Absorption		Distribution	
						log *P*	log *S*	mol/L	HIA (%)	*P*_Caco-2_ (nm/s)	PPB (%)	BBB (*C*_brain_/*C*_blood_)
*Parental ligands*												
ART	282.33	0	5	0	53.99	1.68	− 2.69	0.0020	96.31	30.32	93.36	1.30
ARA	384.42	5	8	1	103.35	1.70	− 1.62	0.0238	83.94	13.21	90.61	0.01
THY	164.20	1	2	0	34.14	2.51	− 2.66	0.0021	99.28	23.03	100.00	1.78
*Artemisinin–thymoquinone hybrids*												
T1	588.69	11	10	0	123.66	4.69	− 6.69	0.0076	97.00	21.18	93.30	0.02
T2	602.71	12	10	0	123.66	5.15	− 7.00	0.0037	97.52	24.18	94.30	0.03
T3	574.66	10	10	0	123.66	4.24	− 6.38	0.0002	96.42	22.11	92.79	0.02
T4	530.65	8	8	0	97.36	4.79	− 6.83	0.0001	99.10	36.05	93.54	0.07
T5	544.68	9	8	0	97.36	5.24	− 7.13	0.0225	99.30	40.17	94.56	0.06
T6	488.61	6	7	0	80.29	4.90	− 6.53	0.0005	99.12	46.47	96.20	0.06
T7	502.64	7	7	0	80.29	5.36	− 6.84	0.0775	99.12	49.14	98.43	0.09
*Control-ligands*												
ANK	509.55	10	9	3	176.34	1.83	− 3.37	0.0004	46.52	17.57	87.45	0.04
AZT	748.996	7	13	5	182.48	2.45	− 3.33	0.0004	69.32	31.40	14.49	0.06
BRT	371.42	4	6	1	120.56	0.40	− 0.14	0.7179	93.54	3.08	78.87	0.00
CLQ	319.88	8	3	1	30.61	4.53	− 4.59	0.0095	98.05	56.61	92.53	7.73
RDS	602.585	14	9	4	203.55	1.71	− 3.18	0.0006	53.50	3.26	81.26	0.04

*AZT* azithromycin, *BRT* baricitinib, *RDS* remdesivir, *ANK* anakinra, *CLQ* chloroquine, *ART* artemisin, *ARA* artemisin acid, *THY* thymoquinone

**Fig. 10:**
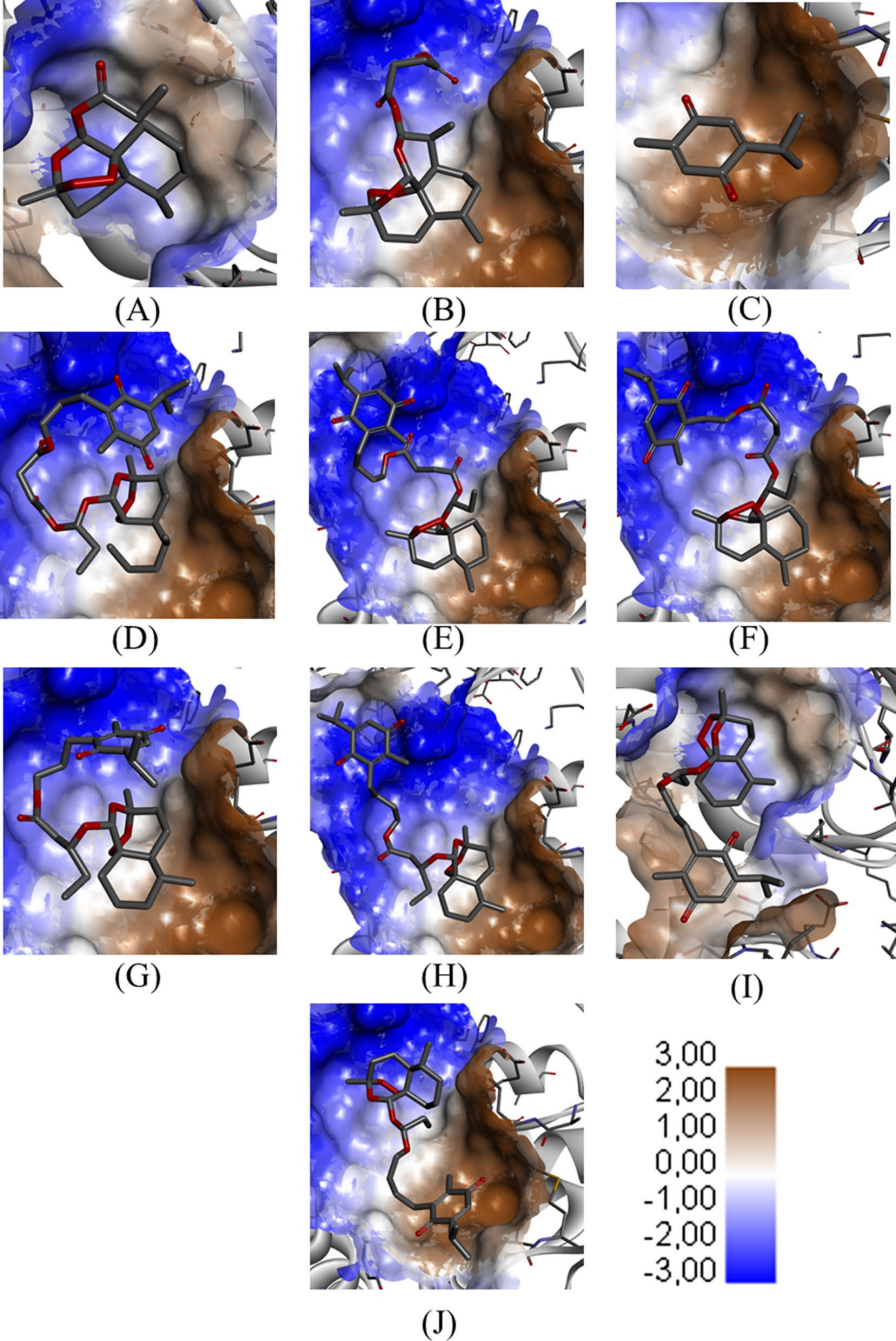
Surface map of ART-THY parental and hybrid compounds with Mpro. **A** Artemisin; **B** artesunic acid; **C** thymoquinone; **D** T1; **E** T2; **F** T3; **G** T4; **H** T5; **I** T6; **J** T7

The structural modifications of the T1–T7 hybrids caused a reduction in their solubility coefficients compared to parental ligands, with emphasis on the substances T1–4 and T6, with solubility values less than 0.01 M. These findings are strongly associated with the values of log *P*, which are increased in hybrids, characterizing these molecules as essentially fat-soluble and, consequently, susceptible to dissolve in the lipid bilayer of most cell membranes. In addition, this pattern is evidenced in the hydrophobicity map illustrated in Fig. 10, in which the regions of interaction vary from lipophilic environments (blue) to hydrophilic environments (brown). In general, these log *P* values were also increased in comparison with the control ligands, except for CLQ, which has lipophilicity similar to hybrids.

### Pharmacokinetic and toxicological study

The pharmacokinetic and toxicological prediction data are shown in Table 2. The lipophilicity and polarity profiles shown demonstrate the ability of the T1–T7 hybrids to penetrate the various biological membranes. In this prediction, the apparent permeability (Papp) of the colorectal adenocarcinoma cell model (Caco-2) of the T1–T3 hybrids below 30 nm/s (Papp < 3 × 10^−6^ cm/s) as well as of the T4–T7 hybrids, where the Papp coefficients are greater than 30 nm/s (Papp > 3 × 10^−6^ cm/s), show their potential permeabilities in the intestinal epithelium, where human intestinal absorptions (HIA) are lower than 97.5% for the first class, and greater than 99% for the second class [40]. In general, these values were higher than those found for the control ligands (less than 70%), except for the assessed absorptions of CLQ (98.05%) and BRT (93.54%).

As for plasma protein binding (PPB), parental ligands showed, in general, high percentage values. In this context, THY stands out, which showed an estimated interaction of 100% of its concentration with plasma proteins, thus presenting an easier distribution in hybridization formed with ART. The PPB values found for the hybrids were higher than those found for the control-ligands, of which only CLQ showed a value of 92.53%; AZT has a PPB of 14.49%.

When the permeability in the blood–brain barrier (BBB) was estimated through the *C*_brain_/*C*_blood_ ratio of the modified chemical entities, an attempt was made to predict the concentration of species in their steady state in the brain and peripheral blood. In the present work, all hybrids showed values of BBB < 0.1, indicating a low passage of these compounds to the central nervous system (CNS). Highly, among the parental ligands, ARA presented BBB exactly equal to 0.1, while ART and THY both had values greater than 1.0. When this parameter was evaluated among the control-ligands, CLQ presented the highest BBB value in the study (7.73), while all the others presented low passage (BBB < 0.1), highlighting BRT which presented the lowest value (< 0.01).

Table 3 lists the characteristics related to metabolism prediction of hybrids, parental and control compounds. When interactions with CYP450 metabolizing enzymes were evaluated, it was observed that T1–T7 products are potential inhibitors of CYP3A4 and CYP2C9, as well as observed for parent compounds. At the same time, AZT, BRT and CLQ, which have been indicated as a potential substrate for metabolism by CYP3A4.

**Table 3:** Metabolism prediction of ART-THY hybrids, parental and control compounds

Compound name	CYP450 inhibition				CYP450 substrate	
	CYP2C19	CYP2C9	CYP2D6	CYP3A4	CYP2D6	CYP3A4
*Parental ligands*						
ART	No	Inhibitor	No	Inhibitor	No	Substrate
ARA	No	Inhibitor	No	Inhibitor	No	Substrate
THY	Inhibitor	Inhibitor	No	Inhibitor	No	Substrate
*Artemisinin–thymoquinone hybrids*						
T1	Inhibitor	Inhibitor	No	Inhibitor	No	Substrate
T2	Inhibitor	Inhibitor	No	Inhibitor	No	Substrate
T3	Inhibitor	Inhibitor	No	Inhibitor	No	Substrate
T4	No	Inhibitor	No	Inhibitor	No	Substrate
T5	Inhibitor	Inhibitor	No	Inhibitor	No	Substrate
T6	No	Inhibitor	No	Inhibitor	No	Substrate
T7	No	Inhibitor	No	Inhibitor	No	Substrate
*Control-ligands*						
ANK	No	No	No	No	No	Weakly
AZT	No	No	Inhibitor	Inhibitor	Weakly	Substrate
BRT	No	No	No	No	No	Substrate
CLQ	No	No	Inhibitor	No	Substrate	Substrate
RDS	No	No	No	Inhibitor	No	Weakly

*CYP450* cytochrome P450, *AZT* azithromycin, *BRT* baricitinib, *RDS* remdesivir, *ANK* anakinra, *CLQ* chloroquine, *ART* artemisin, *ARA* artemisin acid, *THY* thymoquinone

**Fig. 11:**
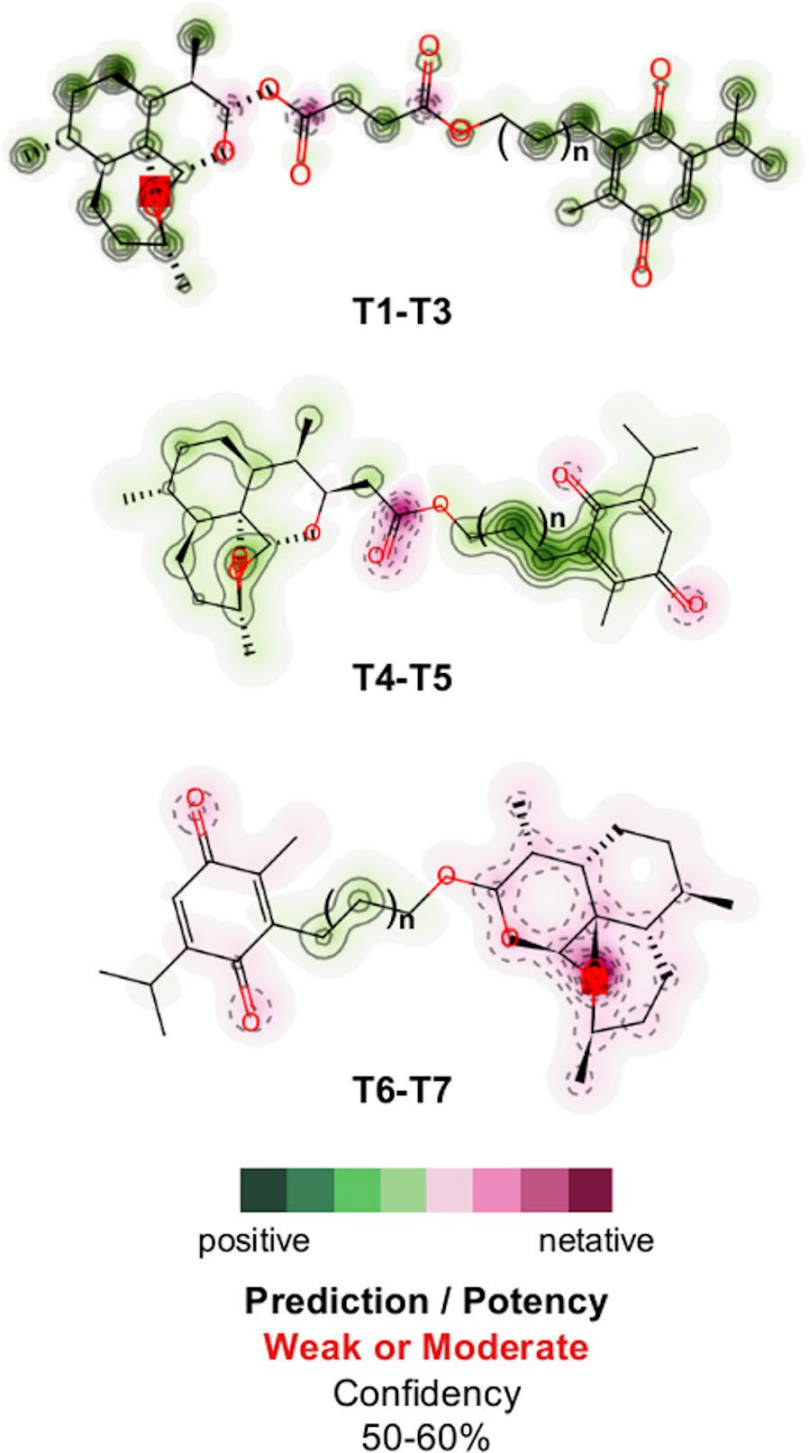
hERG blocking probability map of parental and hybrid compounds (pharmacophore). Positive (green) and negative (pink) structural contributions of T1–T7 hybrids

Finally, Table 4 presents the data related to the prediction of toxicity of the molecules evaluated in the present study. It was observed that none of the hybrids was positive in the test for mutagenicity, in contrast to that observed for ANK, BRT and CLQ. Regarding carcinogenicity, conflicting results were obtained, since the T1–T5 molecules were positive for carcinogenicity in rats and negative in mice. Additionally, the results of inhibition of the hERG channel indicate the low risk of cardiac toxicity of the T1–T7 hybrids, in contrast to the data observed for BRT and CLQ. This trend is structurally observed, as the evaluated QSAR models indicate that synthetic substances have a 50–60% probability of the cardiotoxic effect being weak or moderate. The probability map in Fig. 11 shows that positive structural contributions govern most of the molecular surface of the T1–T5 hybrids, where the negative contributions associated with their carbonyl groups do not pose a cardiotoxic risk. This observation is conflicting in the T6–T7 hybrids, where negative pharmacophores are predominant on their molecular surfaces, with a strong contribution from the carbonyls of the THY fragment and the oxygen atoms of the ART fragment, which may have a cardiotoxic effect. This observation is easily related to the hydrogen interactions between the ligands and the Mpro SARS-CoV-2 receptor, where the carbonyl group is a pharmacophore that constitutes a nucleophilic region of strong interaction between the T1–T3 hybrids with the N^+^H group from the Lys137 residue, as with the T4 hybrid to the Asn238 residue and the T5 hybrid to the Asp197 residue. A special case happens with the T6–T7 hybrids, where the absence of the ester carbonyl groups makes them less susceptible to hydrogen interactions.

**Table 4:** Carcinogenicity and mutagenicity of AMES test and hERG inhibition prediction for the ART-THY hybrids, parental and control compounds

Compound name	Mutagenicity (Ames test)	Carcinogenicity		hERG inhibition
		Mouse	Rat	
*Parental ligands*				
ART	Mutagen	Negative	Positive	Low risk
ARA	Non-mutagen	Negative	Positive	Low risk
THY	Mutagen	Positive	Positive	Low risk
*Artemisinin–thymoquinone hybrids*				
T1	Non-mutagen	Negative	Positive	Low risk
T2	Non-mutagen	Negative	Positive	Low risk
T3	Non-mutagen	Negative	Positive	Low risk
T4	Non-mutagen	Negative	Positive	Low risk
T5	Non-mutagen	Negative	Positive	Low risk
T6	Non-mutagen	Negative	Negative	Low risk
T7	Non-mutagen	Negative	Negative	Low risk
*Control-ligands*				
ANK	Mutagen	Positive	Negative	Ambiguous
AZT	Non-mutagen	Negative	Negative	Ambiguous
BRT	Mutagen	Negative	Negative	Medium risk
CLQ	Mutagen	Negative	Positive	Medium risk
RDS	Non-mutagen	Negative	Negative	Ambiguous

*AZT* azithromycin, *BRT* baricitinib, *RDS* remdesivir, *ANK* anakinra, *CLQ* chloroquine, *ART* artemisin, *ARA* artemisin acid, *THY* thymoquinone

## Discussion

In the present work, it was demonstrated that the hybrids of artemisinin and thymoquinone showed relevant interaction with the active fraction of Mpro enzyme, when compared with reference drugs. Furthermore, hybrids show an improvement in the interaction of substances with the enzyme, mainly due to the higher frequency of interactions with Thr199 residue. When analyzing the physical–chemical properties, it is suggested that hybrids tend to permeate biological membranes, allowing for good human intestinal absorption and with a low partition to central nervous system. Additionally, the hybrids presented themselves as potential inhibitors of CYP-450 enzymes. Finally, none of the hybrids tested positive for mutagenicity and had a low risk of cardiac toxicity.

Theoretical screening studies of pharmacodynamics and pharmacokinetics of synthetic hybrids of artemisinin–thymoquinone with the main protease (Mpro) of SARS-CoV-2 are relevant when aiming to validate their pharmacological activities for the treatment of COVID-19. Several studies describe a better applicability of molecular docking assays using Mpro as a target to potential molecules against SARS-CoV-2, conferring greater specificity when compared to studies involving other enzymes, such as chymotrypsin-like cysteine protease (3CLpro) [5] or NSP10/NSP16 methyltransferase complex [41], used in some studies but belonging to other types of coronavirus.

In the present work, the molecules that showed the most promising results interacted with Mpro at Thr199 amino acid, a different site from that described for the N3 inhibitor, which binds covalently with a Cys145 residue [6]. These findings are important on redirecting of drugs, because from the definition of pharmacophores, it is possible to outline which regions of the receptor are available for efficient binding [25]. The results of in silico studies are evaluated under validated parameters. The N3 complex was used as a standard inhibitor of Mpro in molecular docking studies corroborating previous data showing that the complex is stabilized by multiple hydrophobic interactions and hydrogen bonds. However, pharmacokinetic analyzes show N3 hepatotoxicity and carcinogenicity, confirming the need for other molecules capable of binding to Mpro [42].

In this sense, the hybrids and parental compounds showed characteristics of interaction with the enzyme comparable to those of the control ligands. These comparisons are particularly important for the parameterization and validation of in silico studies. For example, in the work devised by Imberty et al. [38] in which molecular modeling of protein–carbohydrate interactions was performed, relative values for changes in free energy corresponding to the oxygen atoms involved in hydrogen bonds are described, as well as the strength of these bonds based on the distance of the interaction. In their results, the authors describe, for example, interactions with TYR residues in the order of 3.0 Ǻ as strong, corroborating the findings for the hybrids in the present study [38].

However, among the control-ligands, it is important to highlight the results obtained for chloroquine. This drug, which has been used for decades to treat malaria and immune system diseases, came into evidence by inhibiting the replication of SARS Cov-2 in vitro [43]. However, in the past, chloroquine had already shown activity in vitro against several viruses, but without success in animal models or in the treatment of viruses in humans [44, 45]. In the analysis carried out in the present study, CLQ did not show any relevant interaction with the enzyme target, interacting weakly in a different site from the other substances. In addition, CLQ has high lipophilicity, with outstanding permeation and absorption capacity in the intestine, being widely distributed, due to its high binding to plasma proteins and functioning as a potential substrate for metabolism by CYP3A4. These data suggest a good bioavailability, however, CLQ presented the highest BBB value in the study, indicating potential for toxicity in the central nervous system. In addition, this drug showed considerable mutagenic potential and risk of cardiac toxicity.

In fact, chloroquine and hydroxychloroquine are widely used in long-term treatments. However, related cardiac disorder is a rare but severe adverse event, which can lead to death. Nevertheless, there is a lack in the literature related to randomized controlled trials and observational studies. Among the side effect reported, conduction disorders were the main ones. Other non-specific adverse cardiac events included ventricular hypertrophy, hypokinesia, heart failure, pulmonary arterial hypertension and valvular dysfunction [46, 47].

Previous studies using molecular dynamics simulations for the interaction of phenolic compounds and derivatives with Mpro showed that the RMSD values of Mpro remained constant when the simulations were carried out with the bound and unbound complex, strengthening the hypothesis of the high stability of the complexes [42]. Several other studies have been looking for potential active phytochemicals against the Mpro protein, as an in silico molecular dynamics study simulating the interaction of phenolic compounds carried out for a simulation period of 50 ns showed stability of these phytochemicals anchored in the Mpro binding region, associating these findings with the presence of hydrogen bonds since the free-bonding energy analysis, evaluated through the Poisson–Boltzmann surface area (MM-PBSA) shows that the van der Waals energy component has less effect on the bonding affinity. All these findings are correlated with the fact that the radius of rotation of protein–ligand complexes supports their condensed architecture as well as their size [48]. However, there are no data in the literature that perform these analyzes directly with thymoquinones.

Among the hybrids of the present study, a low toxicity is predicted in comparison with the commercially available drugs, considering mainly mutagenicity and cardiotoxicity. In addition, regarding their pharmacokinetic characteristics, it is known that most drugs intended for oral administration are lipophilic and gradually dissolved in the gastrointestinal tract fluid [49]. Together, this information reinforces a possible applicability of hybrids for subsequent pre-clinical and clinical studies.

Additionally, it is known that molecules with high lipophilicity tend to have a high volume of distribution, with low accumulation in the plasma compartment. This, associated with the high rate of binding to plasma proteins, dramatically reduces the fraction of free drug in the plasma, decreasing its speed of hepatic and renal clearance. Thus, it is expected that these molecules have a long half-life, which improves their use orally by reducing the daily number of administrations [50]. This data, associated with the low capacity of hybrids to permeate the blood–brain barrier, gives hybrids the prevention of the occurrence of adverse effects related to the arrival of these substances in the central nervous system [51].

These findings are compatible with the evaluation of the absorption of molecules in the human intestine (HIA) and the evaluation of the permeation of Caco-2 cells, concluding that the hybrids are compounds with high absorption and moderate permeability in Caco-2 cells, which can vary from 20 to 70% [52, 53]. This information is associated with the obtained log *P* values, indicating that these molecules may have the ability to penetrate cells and, consequently, have direct contact with the target enzyme [54].

In addition, T1–T7 tend to have a better distribution of their available fractions to carry out diffusion and transport across cell membranes, as well as increase the energy of interaction with the SARS-CoV-2 target. It is important to mention that only the fraction of the drug not bound to plasma proteins is easily subject to these series of interactions described here [36]. In the present study, it was observed that the substances T1–T7 are strongly bound to plasma proteins, but with a fraction available to interact with the SARS-CoV-2 target compared to THY, which showed an estimated protein binding of 100%.

Regarding the metabolization of the studied hybrids, an affinity with CYP3A4 and CYP2C9 enzymes is observed, as well as for the parent compounds. This indicates the possibility of drug interactions, causing a reduction in the rate of metabolism of drugs such as AZT, BRT and CLQ, which were also indicated as a substrate for metabolism by CYP3A4. This information suggests the possibility of an association between the hybrids and one of the drugs mentioned, in order to increase the half-life and generate a possible synergistic effect [55].

Considering drug metabolism by CYP3A4, an enzyme that promotes reactions such as hydroxylation, aromatic and heteroatom oxidation, in addition to N- and O-dealkylations, the emergence of drug interactions with several drugs used for a variety of diseases is possible. However, considering the desired application for the treatment of COVID-19 infection, the use of the molecules presented here for short periods should be taken into consideration, which minimizes this risk [56].

In general, T1–T7 hybrids present structural contributions in the form of pharmacophores that determine their biological activity. The carbonyl groups of the T1–T5 structures constitute highly attractive nucleophilic hydrogen bond receptor regions with the N^+^H charged groups of specific residues of the Mpro SARS-CoV-2 receptor, such as Lys137, Asp197 and Asn238, characteristic of the site of activity of the drugs ANK, AZT, BRT and RDS [57]. However, the low susceptibility to hydrogen bonding of the T6 hybrid directed the ligand to a distinct region on the receptor, reflecting its synergistic activity associated with the control ligands.

## Conclusions

In conclusion, the adversity surrounding the COVID-19 pandemic has reached a global scale and, with this advance, there is a need for theoretical and medicinal Chemistry, as well pharmacological professionals to develop new drugs with significant anti-SARS-CoV-2 replication. The results presented here demonstrated that the hybrid products of artemisinin and thymoquinone present interaction with Mpro, with desirable characteristics of pharmacokinetics and toxicity compared to the drugs available on the market. Thus, these molecules are promising candidates for the development of specific drugs against COVID-19.

## Data Availability

All data generated or analyzed during this study are included in this published article.

## Abbreviations

∆*G*: Binding free energy
ADME: Absorption, distribution, metabolism and excretion
ANK: Anakinra
ARA: Artemisin acid
ART: Artemisin
AZT: Azithromycin
BBB: Permeability through the blood–brain barrier
BRT: Baricitinib
CLQ: Chloroquine
CYP450: Cytochrome P450
Cys: Cysteine
HBA: Number of H-bond acceptors
HBD: Number of H-bond donors
HCMV: Human cytomegalovirus
hERG: Human Ether-a-go-go-Related Gene
HIA: Human intestinal absorption
MMFF94: Merck Molecular Force Field 94
Mpro: Main protease
MW: Molecular mass
PPB: Plasma protein binding
RB: Rotating bonds
RDS: Remdesivir
SARS-CoV-2: Severe acute respiratory syndrome coronavirus 2
SARS: Severe acute respiratory syndrome
Ser: Serine
THY: Thymoquinone
TPSA: Topological polar surface area
WHO: World Health Organization
